# Supplementary material for: The EV71 2A protease occupies the central cleft of SETD3 and disrupts SETD3-actin interaction
Source: Nat Commun. 2024 May 16;15:4176. doi: 10.1038/s41467-024-48504-w (PMC11099015; doi:10.1038/s41467-024-48504-w)
Supplement: Supplementary file 1 — Supplementary Information [file 41467_2024_48504_MOESM1_ESM.pdf]

Supplementary Materials for

The EV71 2A protease occupies the central cleft of SETD3 and disrupts SETD3-actin interaction

Supplementary Figures

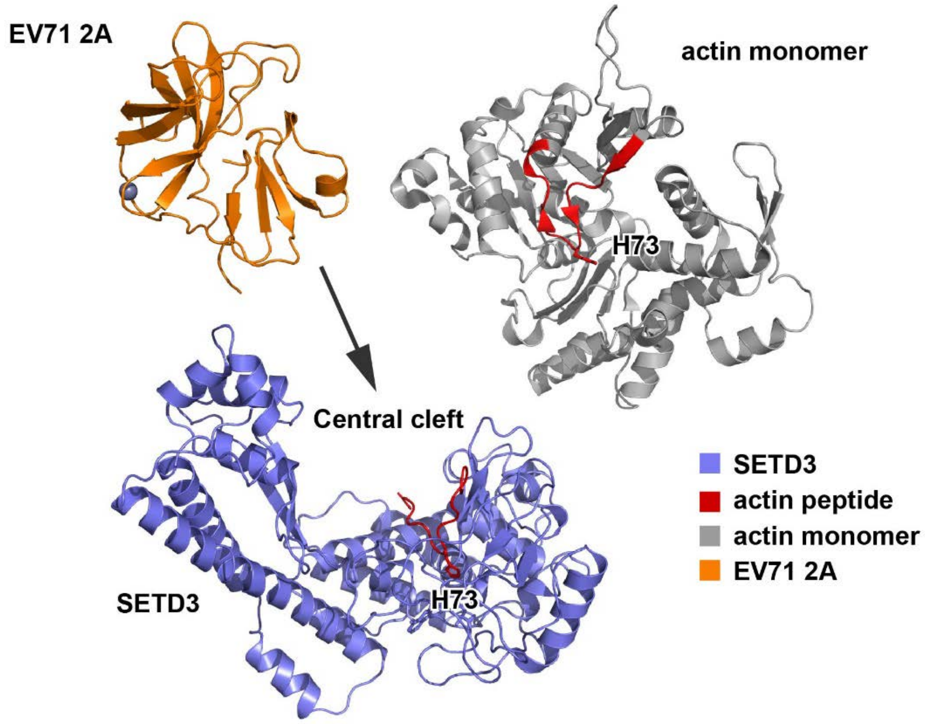

Supplementary Figure 1 Comparison of size and shape among SETD3, EV71 2A and actin monomer.

Comparing structures of SETD3 (PDB: 6MBK), EV71 2A (PDB: 3W95) and actin monomer (PDB: 5OOF) suggests that SETD3 central cleft is too narrow for an actin monomer, and dramatic conformational change of actin must occur to gain access into the SETD3 active site. By contrast, the size of EV71 2A suggests it might fit into the SETD3 central cleft, which implies 2A could disrupt the SETD3-actin complex via competitive binding. The actin peptide complexed by SETD3 and the same region its original conformation in actin is highlighted in red.

a

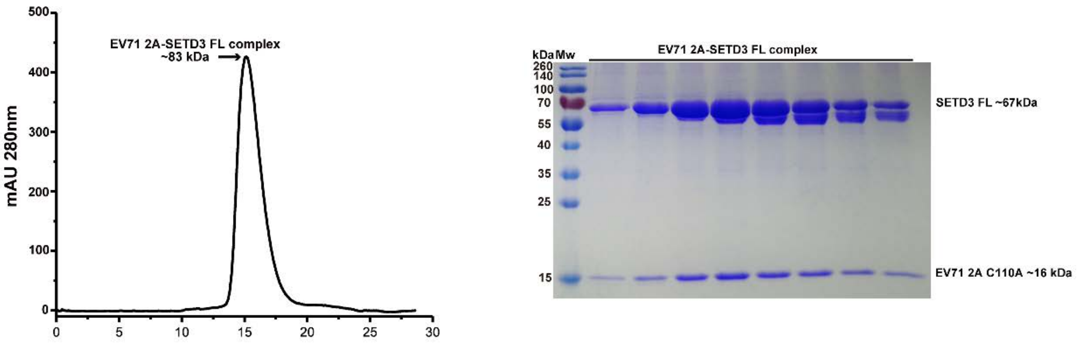

b

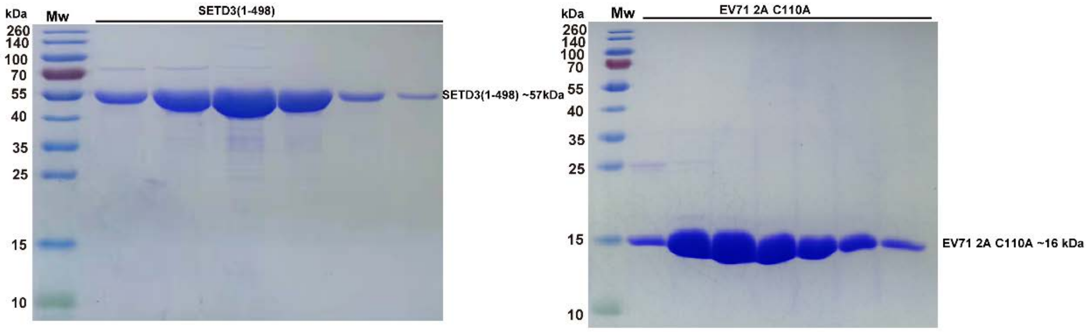

Supplementary Figure 2. Purification of SETD3 variants and EV71 2A C110A mutants for structural and biochemical studies.

a) Full-length SETD3 was co-expressed with EV71 2A C110A mutant (a catalytically inactive mutant) in insect cells to prepare protein complexes. The SDS-PAGE analysis of the co-expressed proteins demonstrate that SETD3 FL was less stable than SETD3(1-503) and SETD3(1-498). Data are representative of three independent experiments. Source data are provided as a Source Data file.

b) To prepare complex for crystallization, SETD3 truncation 1-498 aa and EV71 2A C110A were separately expressed and purified. The SDS-PAGE gel shows the purity of SETD3 1-498 aa and EV71 2A C110A eluted from a gel filtration column. Data are representative of three independent experiments. Source data are provided as a Source Data file.

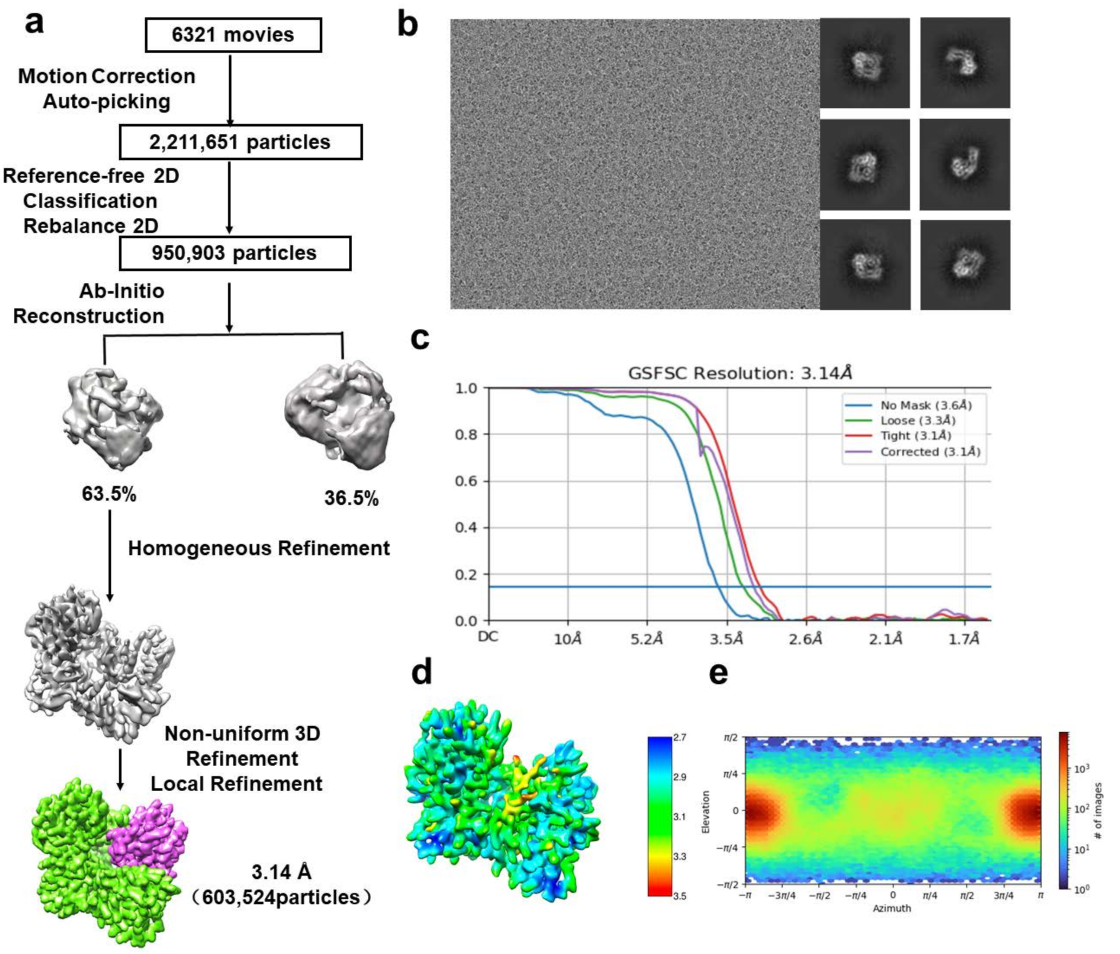

Supplementary Figure 3. Cryo-EM data analysis.

a) Workflow of Cryo-EM data processing for SETD3 1-503 (insect cell expressed) complexed with EV71 $2A^{pro}$ C110A.

b) Left, representative micrograph of the SETD3 (1-503) - EV71 $2A^{pro}$ complex; representative classes of a 2D classification of the particles used for final reconstruction of the complex.

c) Gold-standard Fourier shell correlation (FSC) curves of the final SETD3 (1-503) - EV71 $2A^{pro}$ complex reconstruction. The blue line indicates the 0.143 cutoff criterion, indicating a nominal resolution of 3.14 Å.

d) Local resolution calculated using cryoSPARC, blue to red indicates high to low resolution.

e) Angular distribution of particles used in the final reconstruction.

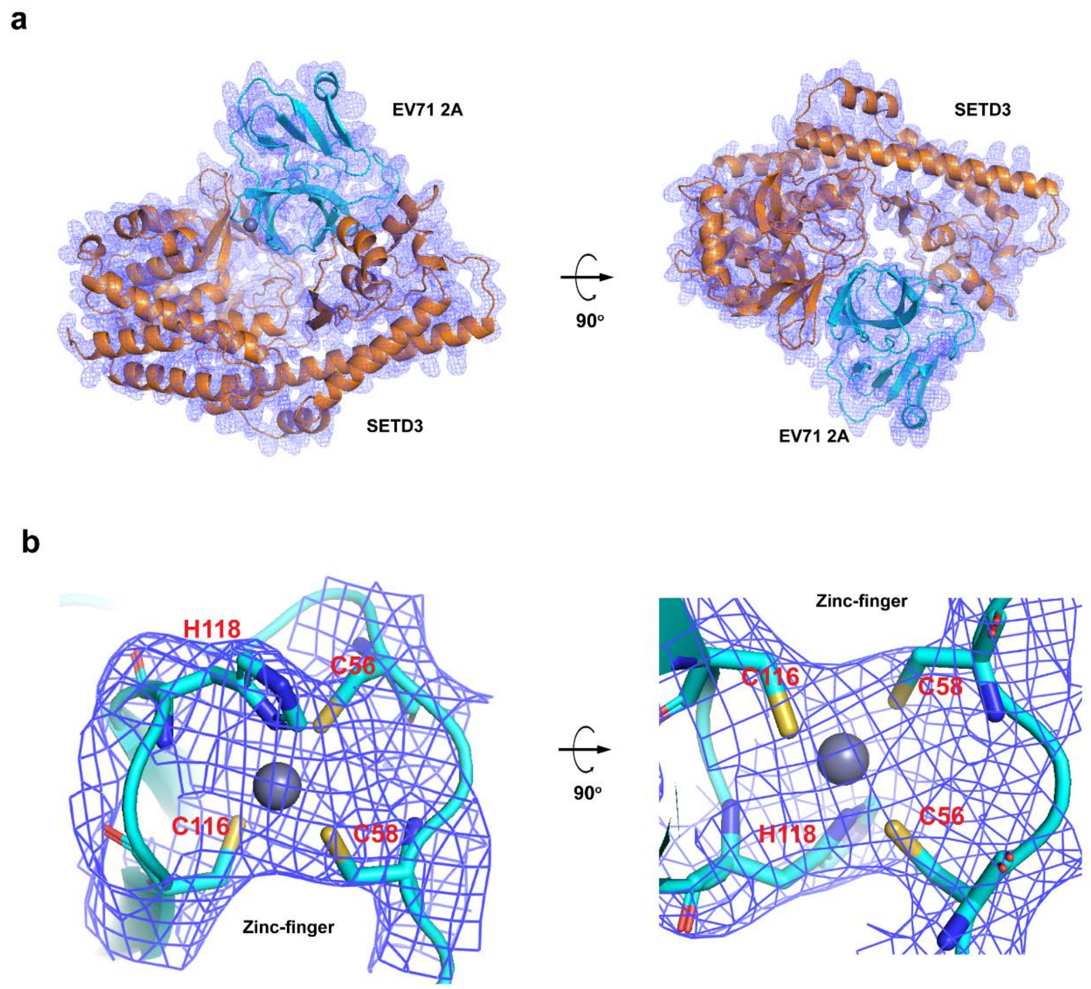

Supplementary Figure 4. EM densities for the SETD3 in complex with EV71 2A

a) Overall density map for SETD3-EV71 2A complex. EV71 2A in cyan and SETD3 in orange. Right: the same model rotated around the vertical axis by 90°

b) Cryo-EM density map (blue mesh) of the EV71 2A zinc-finger. Right: the same model rotated around the vertical axis by 90°

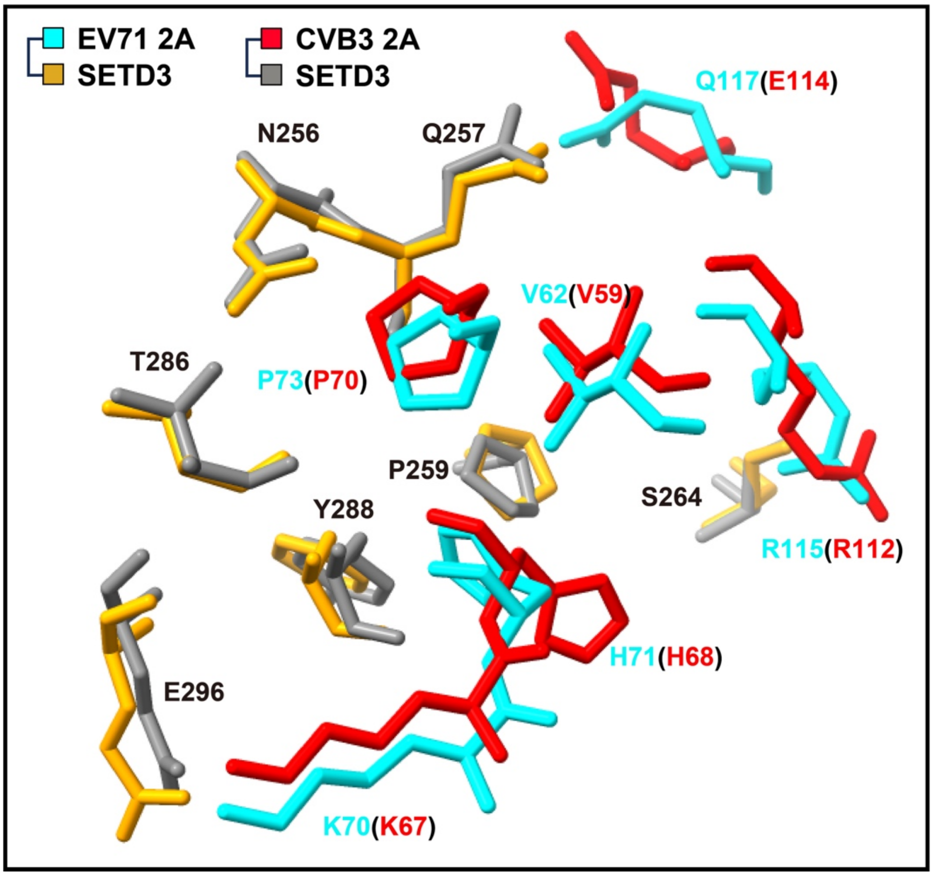

Supplementary Figure 5. Superimposition of structures of the SETD3-EV71 2A and the SETD3-CVB3 2A complexes reveal conserved interfacial residues.

The structures of SETD3 (gold)-EV71 2A (cyan) and the SETD3 (gray)-CVB3 2A (red) complexes are superimposed. Residues at SETD3-2A interface are shown with the stick models with labels.

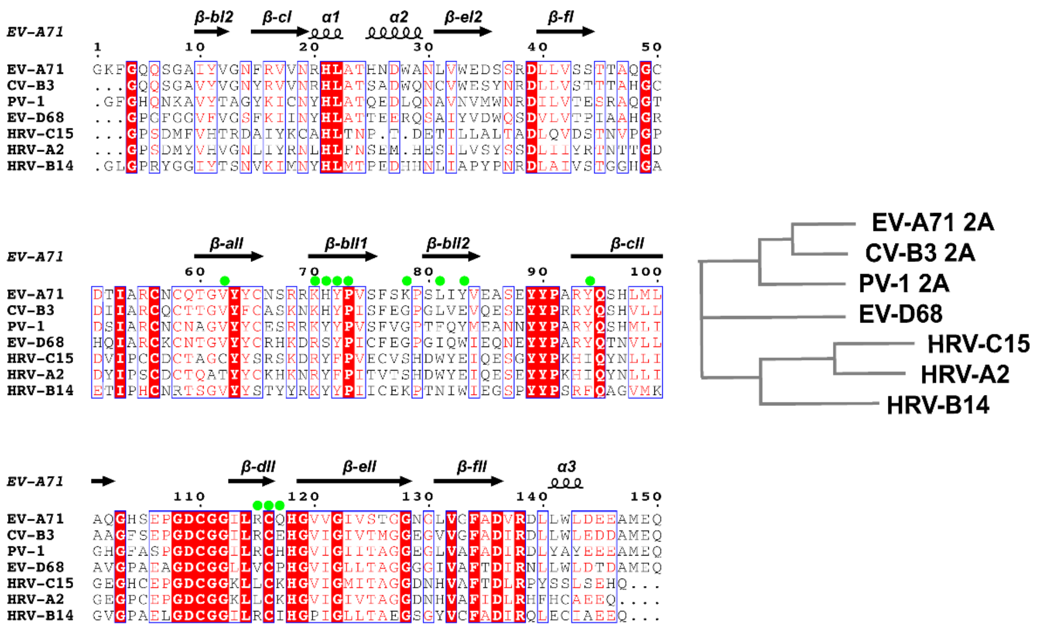

Supplementary Figure 6. Structure-based multiple sequence alignment of various enterovirus 2A proteases

The EV71 2A, CVB3 2A, PV 2A, EV-D68 2A, and three different HRV 2As were used. Secondary structural elements of EV71 2A are aligned on top of the sequences. Residues involved in direct interaction with SETD3 are highlighted by green dots. Right insert, phylogenetic tree of various enteroviral 2A sequences generated by Clustal Omega.

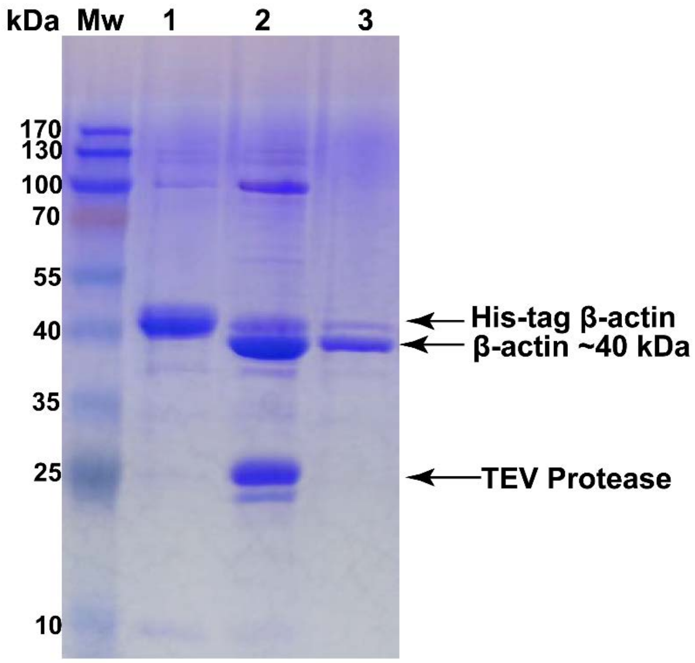

Supplementary Figure 7. Expression and purification of human β-actin

Lane 1, His-tag β-actin eluate from the Ni-NTA resin; lane 2, digestion by TEV overnight; lane 3, flow through from the Ni-NTA column. While the cleaved His tag and undigested His-tag β-actin species were trapped on the column, non-tagged-β-actin proteins were collected. Data are representative of three independent experiments. Source data are provided as a Source Data file.

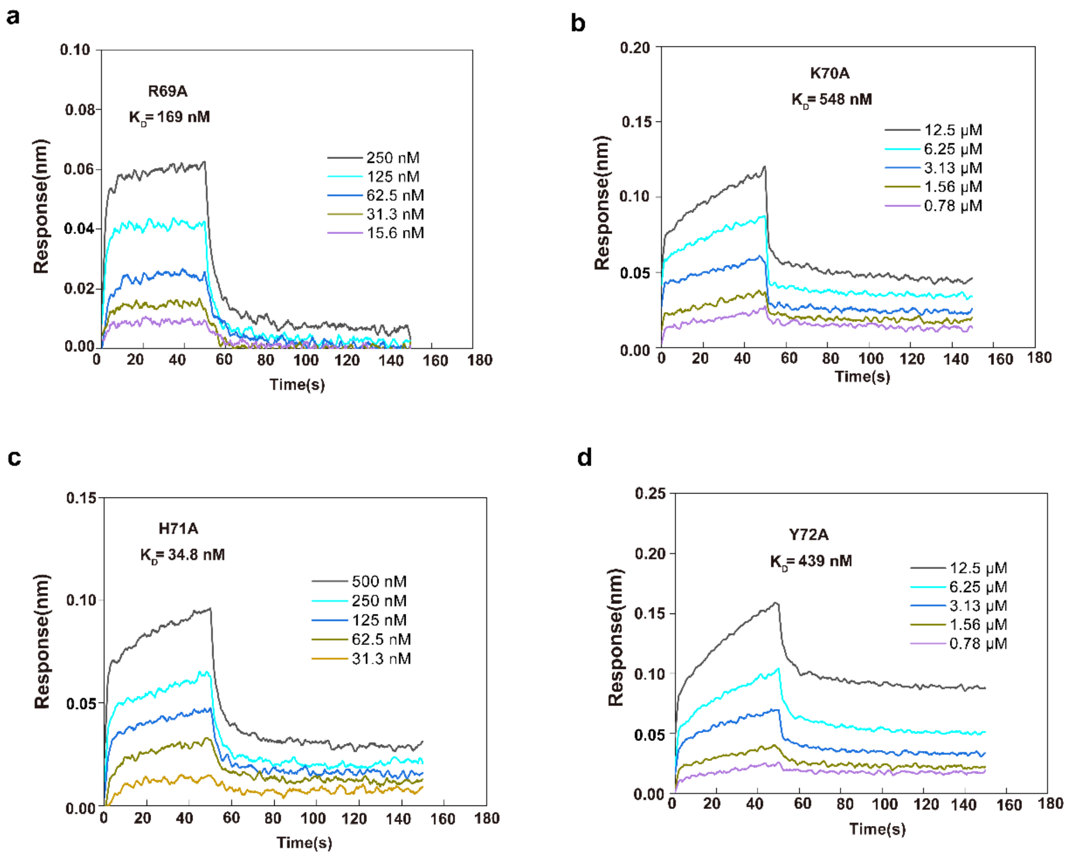

Supplementary Figure 8. BLI assay between EV71 2A mutants and SETD3

a-d. BLI sensorgrams for the interaction between EV71 2A mutants in addition to C110A at different concentrations was exposed to SETD3. The equilibrium constants K_d values are indicated.

Supplementary Tables

Supplementary Table 1

Data collection and refinement statistics.

	**EV71 2A C110A in complexed SETD3 (PDB ID: 8X77)**
**Data collection**	
Space group	$P2_1$
Cell dimensions	
a, b, c (Å)	149.00 57.49 198.76
α, β, γ (°)	90.00, 110.93, 90.00
X ray source	
Wavelength (Å)	0.98
Data range (Å)	49.34-3.50
Reflections unique	76,091[a]
R_{sym} [b] (highest resolution shell)	0.614 (2.099)
$I / \sigma I$ (highest resolution shell)	2.29 (0.6)
Completeness (%) (highest resolution shell)	97.9 (92.3)
Redundancy (highest resolution shell)	3.51 (3.59)
**Refinement**	
Resolution range (Å)	19.99-3.52
Reflections	39,534[a]
R_{work} [c]/ R_{free} [d] (highest resolution shell)	0.2739/0.2914 (0.2766/ 0.3214)
**Atoms**	
Non-hydrogen protein atoms	21033
Protein	19481
Solvent	1526
B-factors average (Å^2)	58.99
Protein (Å^2)	59.03
Ligands (Å^2)	0
Solvent (Å^2)	56.75
**r.m.s.d**	
Bond lengths (Å)	0.013
Bond angles (°)	0.995
% residues in favored regions, allowed regions, outliers in Ramachandran plot	96.30,3.62,0.08

Values in parentheses are for the highest-resolution shell.

[a] Friedel pairs are treated as different reflections

[b] $R_{sym} = \sum_{hkl}\sum_j |I_{hkl,j} - I_{hkl}|/\sum_{hkl}\sum_j I_{hkl,j}$, where I_{hkl} is the average of symmetry-related observations

of a unique reflection

[c] $R_{work} = \sum_{hkl} ||F_{obs}(hkl)|-|F_{calc}(hkl)||/\sum_{hkl}|F_{obs}(hkl)|$.

[d] R_{free}= the cross-validation R factor for 5% of reflections against which the model was not refined.

Supplementary Table 2

Cryo-EM data collection parameters, refinement and validation statistics on reconstruction and the SETD3-2A model

**Data collection**	
Microscope	FEI Titan Krios
Voltage (keV)	300
Nominal Mag	29000x
Exposure navigation	Stage position/beam and image shift
Cumulative dose (e/Å^2)	66
Requested defocus range (um)	-1.8-2.5
Detector Gatan	K3
Pixel size (physical pixel, Å)	0.82
Dose rate (e^{-}/physical pixel/sec)	8
Total exposure time (sec)	6
Micrographs collected	6321
**Reconstruction**	
Initial particles used	2,211,651
Particles selected after 2D classification	950,903
Particles used in final 3D reconstruction	603,524
Symmetry Imposed	C1
Map Res (Å), masked/unmasked	3.1/3.6
FSC Threshold	0.143
Resolution range (local), Å	3.03-3.14
Final bfactor applied	-60
**Model Refinement**	
Initial Model (PDB)	3W95 6MBJ
Protein residues (atoms)	622(4949)
Ligands (atoms)	1(1)
Map Correlation Coefficient (masked)	0.87
RMSD, Bond Lengths (Å)	0.004
RMSD, Bond Angles (°)	0.825
**Validation**	
Ramachandran Outliers (%)	0
Ramachandran Allowed (%)	1.46
Ramachandran Favored (%)	98.54
MolProbity score	1.83
Clashscore (all atoms)	21.86
Rotamer outliers (%)	0.57

Supplementary Table 3

Actin synthetic peptide used in this study

Peptide #	Peptide name	Sequence (N’ to C’)
**1**	Short actin（66-80）	**TLKYPIEHGIVTNWD**

Supplementary Table 4

Comparison of kinetics of SETD3 on H73(66–80) peptide in presence of different concentrations of 2A.

Protein	$k_{cat}(h^{-1})$	$K_m(\mu M)$
**SETD3 Alone**	**18.5±0.3**	**8.1±0.5**
**SETD3:2A (1:2)**	**13.5±1.0**	**38.9±6.8**
**SETD3:2A (1:5)**	**9.6±0.8**	**44.9±8.8**
