# Supplementary material for: The EV71 2A protease occupies the central cleft of SETD3 and disrupts SETD3-actin interaction
Source: Nat Commun. 2024 May 16;15:4176. doi: 10.1038/s41467-024-48504-w (PMC11099015; doi:10.1038/s41467-024-48504-w)
Supplement: Supplementary file 3 — Description of Additional Supplementary Files [file 41467_2024_48504_MOESM3_ESM.pdf]

Description of Additional Supplementary Files:

Supplementary Data 1: Optimized genes in this study

Supplementary Data 2: List of primers used in this study
