# Supplementary material for: The EV71 2A protease occupies the central cleft of SETD3 and disrupts SETD3-actin interaction
Source: Nat Commun. 2024 May 16;15:4176. doi: 10.1038/s41467-024-48504-w (PMC11099015; doi:10.1038/s41467-024-48504-w)
Supplement: Supplementary file 6 — Reporting Summary [file 41467_2024_48504_MOESM6_ESM.pdf]

nature portfolio

Corresponding author(s): Sheng CUI

Last updated by author(s): Dec 18, 2023

Reporting Summary

Nature Portfolio wishes to improve the reproducibility of the work that we publish. This form provides structure for consistency and transparency in reporting. For further information on Nature Portfolio policies, see our Editorial Policies and the Editorial Policy Checklist.

Statistics

For all statistical analyses, confirm that the following items are present in the figure legend, table legend, main text, or Methods section.

n/a	Confirmed	
[ ]	[x]	The exact sample size (n) for each experimental group/condition, given as a discrete number and unit of measurement
[ ]	[x]	A statement on whether measurements were taken from distinct samples or whether the same sample was measured repeatedly
[x]	[ ]	The statistical test(s) used AND whether they are one- or two-sided<br>*Only common tests should be described solely by name; describe more complex techniques in the Methods section.*
[x]	[ ]	A description of all covariates tested
[x]	[ ]	A description of any assumptions or corrections, such as tests of normality and adjustment for multiple comparisons
[ ]	[x]	A full description of the statistical parameters including central tendency (e.g. means) or other basic estimates (e.g. regression coefficient) AND variation (e.g. standard deviation) or associated estimates of uncertainty (e.g. confidence intervals)
[x]	[ ]	For null hypothesis testing, the test statistic (e.g. F, t, r) with confidence intervals, effect sizes, degrees of freedom and P value noted<br>*Give P values as exact values whenever suitable.*
[x]	[ ]	For Bayesian analysis, information on the choice of priors and Markov chain Monte Carlo settings
[x]	[ ]	For hierarchical and complex designs, identification of the appropriate level for tests and full reporting of outcomes
[x]	[ ]	Estimates of effect sizes (e.g. Cohen's d, Pearson's r), indicating how they were calculated

Our web collection on statistics for biologists contains articles on many of the points above.

Software and code

Policy information about availability of computer code

Data collection	BL19U1 beamline of National Facility for Protein Science in Shanghai (NFPS), the staff of the BL17U1 beamline and the staff of the BL10U2 beamline at the Shanghai Synchrotron Radiation Facility for assistance in data collection.<br>SerialEM software<br>LI-COR Odyssey collect the western blot data<br>SpectraMax M5<br>ForteBio Octet RED96e<br>Beckman Optima XL-I
Data analysis	cryoSPARC-3.3.2, MotionCor2 1.2.2, CTFFIND 4.1.13, UCSF Chimera 1.14, ChimeraX 1.4, Phenix 1.17.1, Coot 0.8.9.2 EL, PyMOL 2.3.4 , ;XDS package,CCP4i,Graphpad Prism version 8; Image J version 1.8.0;Origin Version 9,

For manuscripts utilizing custom algorithms or software that are central to the research but not yet described in published literature, software must be made available to editors and reviewers. We strongly encourage code deposition in a community repository (e.g. GitHub). See the Nature Portfolio guidelines for submitting code & software for further information.

Data

Policy information about availability of data

All manuscripts must include a data availability statement. This statement should provide the following information, where applicable:

- Accession codes, unique identifiers, or web links for publicly available datasets
- A description of any restrictions on data availability
- For clinical datasets or third party data, please ensure that the statement adheres to our policy

The 3D cryo-EM density map of the SETD3(1-503)-EV71 2A complex has been deposited in the EM Database under the accession codes EMD-38156, and the coordinate for the structure have been deposited in Protein Data Bank under accession code 8X8Q [https://doi.org/10.2210/pdb8X8Q/pdb]. The atomic coordinates and structure factors for SETD3(1-498)-EV71 2A complex have been deposited in the Protein Data Bank under the accession codes: 8X77 [https://doi.org/10.2210/pdb8X77/pdb]. Publicly available protein atomic models with the following PDB code were used in the study: 6MBK[https://doi.org/10.2210/pdb6MBK/pdb],3W95[https://doi.org/10.2210/pdb3W95/pdb],5OOF[https://doi.org/10.2210/pdb5OOF/pdb],7LMS[https://doi.org/10.2210/pdb7LMS/pdb],6OX2 [https://doi.org/10.2210/pdb6OX2/pdb] ,3SMT[https://doi.org/10.2210/pdb3SMT/pdb] and 4FVD[https://doi.org/10.2210/pdb4FVD/pdb]. Source data are provided with this paper.

Research involving human participants, their data, or biological material

Policy information about studies with human participants or human data. See also policy information about sex, gender (identity/presentation), and sexual orientation and race, ethnicity and racism.

Reporting on sex and gender	N/A
Reporting on race, ethnicity, or other socially relevant groupings	N/A
Population characteristics	N/A
Recruitment	N/A
Ethics oversight	N/A

Note that full information on the approval of the study protocol must also be provided in the manuscript.

Field-specific reporting

Please select the one below that is the best fit for your research. If you are not sure, read the appropriate sections before making your selection.

☒ Life sciences ☐ Behavioural & social sciences ☐ Ecological, evolutionary & environmental sciences

For a reference copy of the document with all sections, see nature.com/documents/nr-reporting-summary-flat.pdf

Life sciences study design

All studies must disclose on these points even when the disclosure is negative.

Sample size	603,524 particles from 6,321 micrographs were used for 3D reconstruction of EV71-2A and SETD3 complex. These cryo-EM images were sufficient to determine the structure of satisfactory quality. We conducted three replicates for the functional assays, each of which was successful.
Data exclusions	The date sets for crystal diffraction were merged based on completeness,R-factor,I/sigma.<br>Following multiple rounds of 2D and 3D classification, particles that did not meet the desired class criteria or showed significant flaws based on established cryo-EM principles were removed from the cryo-EM analysis.
Replication	All functional assays were carried out independently at least three times ,and all attempts at replication were successful.
Randomization	This study did not include any statistical comparisons.
Blinding	Blinding was not considered at current state.For cryo-EM and crystallography, data were collected automatically and data processing is also implemented automatically by the software.

Reporting for specific materials, systems and methods

We require information from authors about some types of materials, experimental systems and methods used in many studies. Here, indicate whether each material, system or method listed is relevant to your study. If you are not sure if a list item applies to your research, read the appropriate section before selecting a response.

Materials & experimental systems

n/a	Involved in the study
	☒ Antibodies
	☒ Eukaryotic cell lines
☒	☐ Palaeontology and archaeology
☒	☐ Animals and other organisms
☒	☐ Clinical data
☒	☐ Dual use research of concern
☒	☐ Plants

Methods

n/a	Involved in the study
☒	☐ ChIP-seq
☒	☐ Flow cytometry
☒	☐ MRI-based neuroimaging

Antibodies

Antibodies used

The following antibodies were used in this study: β-actin (A1978; Sigma; Dilution 1:5000), V5 (V8012; Sigma; Dilution 1:1000), EV71-VP1 (MAB1255-M05; Abnova; Dilution:1:1000), and SETD3 (ab176582; Abcam; Dilution 1:2000). IRDye 680- and 800-labeled secondary antibodies were purchased from LI-COR Biosciences (926-68020, 926-32211, 926-68073; Dilution: 1:5000-1:20000). Anti-EV71 2A antibody was generated in rabbits using recombinant protein as the immunogen (Dilution 1:5000).

Validation

The applications of all antibodies used in this study were verified either by the manufacturers or by our laboratory. All validation statements are available on the manufacturer's website.
β-actin (A1978; Sigma; Dilution 1:5000):https://www.sigmaaldrich.cn/CN/en/product/sigma/a1978
V5 (V8012; Sigma; Dilution 1:1000):https://www.sigmaaldrich.cn/CN/en/product/sigma/v8012
EV71-VP1 (MAB1255-M05; Abnova; Dilution:1:1000):https://www.abnova.com/en-global/product/detail/mab1255-m05
SETD3 (ab176582; Abcam; Dilution 1:2000):It has been discontinued now. We have verified that this alternative can be used.https://www.abcam.com/products/proteins-peptides/recombinant-human-setd3-protein-ab132885.html
IRDye 680- and 800-labeled secondary antibodies were purchased from LI-COR Biosciences (926-68020, 926-32211, 926-68073; Dilution: 1:5000-1:20000):https://www.licor.com/bio/reagents/irdye-680lt-goat-anti-mouse-igg-secondary-antibody;https://www.licor.com/bio/reagents/irdye-800cw-goat-anti-rabbit-igg-secondary-antibody;https://www.licor.com/bio/reagents/irdye-680rd-donkey-anti-rabbit-igg-secondary-antibody

Eukaryotic cell lines

Policy information about cell lines and Sex and Gender in Research

Cell line source(s)

high-five cells (Invitrogen) , RD cells(ATCC), BSR/T7(Laboratory preserved)

Authentication

high-five cells were authenticated by Invitrogen and RD cells were authenticated by ATCC

Mycoplasma contamination

All cell lines were mycoplasma-free

Commonly misidentified lines
(See ICLAC register)

N/A

Plants

Seed stocks

N/A

Novel plant genotypes

N/A

Authentication

N/A
